# Supplementary material for: Intraspecific host variation plays a key role in virus community assembly
Source: Nat Commun. 2020 Nov 5;11:5610. doi: 10.1038/s41467-020-19273-z (PMC7644774; doi:10.1038/s41467-020-19273-z)
Supplement: Supplementary file 1 — Supplementary Information [file 41467_2020_19273_MOESM1_ESM.pdf]

Sallinen *et al*. Supplementary information

Supplementary table 1. Detailed information of the five studied viruses. Information marked with an asterisk* is based on viruses in the same family or genus, as reported in ICTV reports

Virus	Family / Genus	Genome type	Genome size	Vectors	Transmission	Known host range	References
Plantago lanceolata latent virus	Geminiviridae / Capulavirus	ssDNA	2.8 kbp	Aphid *, mechanical (-)	Persistent, circulative *	Experimental inoculations to other hosts successful, but wild infections only found in *P.lanceolata*	Romagnac *et al.* 2015[1], Susi et al. 2017[2]
Plantago latent caulimovirus	Caulimoviridae / Caulimovirus	dsDNA	7.2-9.2 kbp *	Aphids *, mechanical	Semi-persistent*	Narrow, single plant family. Dicotyledons. *	Susi et al. 2019[3]
Plantago closterovirus	Closterviridae / Closterovirus	ssRNA (+)	14.5–19.3 kbp *	Varies: Aphids, whiteflies, pseudococcid mealybugs and soft scale insects, grafting. Possibly through SAP, but not by seed. *	Semi-persistent*	Mostly herbaceous plants, berry crops, fruit crops. *	Susi et al. 2019[3] Fuchs et al. 2020 [4]
Plantago enamovirus	Luteoviridae / Enamovirus	ssRNA(+)	5.6-6.0 kbp *	Aphids, mechanical *	Circulative, non-propagative *	NA	Susi et al. 2019[3] ICTV[5]
Plantago betapartitivirus	Partitiviridae / Betapartitivurs	dsRNA	3-4.8 kbp *	No known vectors, cell-division*	Cell-divition*	NA	Susi et al. 2019[3] Vainio et al. 2018[6]

Supplementary methods

PCR detection of viruses

Detection of viruses from *Plantago lanceolata* DNA and cDNA samples were performed following the protocol described in Susi et al. (2019). PCR reactions to detect viruses were performed in final volume of 10 µL containing of 1-3 µL of DNA or cDNA (S2), and GoTaq Green® polymerase 5x Mastermix (Promega Corporation, USA) according to manufacturer's instructions. Samples were subjected to initial denaturation in 95 °C for 2 min, following 35 cycles of denaturation in 95 °C for 40 s, annealing period of 53–60 °C for 40 s (Table S2), and extension 72 °C for 1 min with a final extension step of 72 °C for 5 min. The amplicons were resolved on a 1.2-1.5% agarose gel and visualized using Gel Doc XR System (Bio-Rad Laboratories, Inc., USA).

Supplementary table 2. Virus specific information on primers, product size and annealing temperatures.

Virus	Forward primer	Reverse primer	Product size (nt)	Annealing tm	Reference
Plantago latent caulimovirus	TGAAGTTTTTATTATCCGTTCGTACC	CAAAGCAAATAAAGGAATTACTTGACC	1,089	50	Susi et al. 2019
	AGGAGATGCCCATACTTTACC	GACTTGCCAGAACCTGATTTAC	100	60	This study
Plantago lanceolata latent virus	CAGTCCACACTTCCGCAGTA	AACCACACCACCCCAATATC	607	52	Susi et al. 2017
	GTGTTTAACAATGAAGTGAGCC	AATCCATCCACACATCCAATC	117	60	This study
Plantago betapartitiviru s	TCCGTCCTGTTTATGCTGTTGA	TCTTGCAGACATGTGAGGC	974	53	Susi et al. 2019
Plantago enamovirus	GGCTGGCCAAAGAAGGGG	GCCAGGTTAGTCGACGTGCTCT	929	57	Susi et al. 2019
Plantago closterovirus	GATTTACCCCAGAACTGTTGGGTG	CTAACTTCTTCAGTTAAAGCGCGAGAA	790	50	Susi et al. 2019

Supplementary results for the co-occurrence analysis

We analysed the co-occurrence patterns both in the full data set as well as separately for each host genotype and plant population with the R package ‘cooccur’ [7] and its identically named function. The algorithm applies a probabilistic model [8], which calculates expected frequencies of species co-occurrences based on a distribution of random, independent species. By comparing the expected and observed co-occurrences this analysis gives the probabilities of co-occurrence greater than or less than what is observed in the data. As a result, we get probabilities for the co-occurrence of two species at a frequency either less or greater than the observed frequency of co-occurrence, under the condition that a species probability of occurrence at each site is equal to its observed frequency among all the sites. The results can be interpreted and reported as p-values, without reference to a statistic, so that for a given alpha level, say 0.1, a P(less) < 0.1 suggests that those species are negatively associated, or correspondingly P(greater) < 0.1 suggests that those species are positively associated.

Supplementary table 3. Results for the co-occurrence analysis presented in Figure 2 in the main text.

Data	Species 1	Species 2	Expected number of co-occurrences	Observed number of co-occurrences	P(*lt*): Probability of co-occurrence at a frequency *less* than the observed frequency	P(*gt*): Probability of co-occurrence at a frequency *greater* than the observed frequency
Full data	*Be*	*Cau*	3.2	6	0.986	0.0596
	Cap	*Cau*	2.6	6	0.996	0.0232
Host genotype 609_19	*Be*	*Cau*	2.0	4	0.985	0.0957
Host genotype 2818_6	*Clo*	*Be*	6.9	4	0.0975	1.000
	Clo	*Cap*	2.2	0	0.0639	1.000
	Be	*Cap*	1.9	4	0.985	0.0861
Local population 433	*Clo*	*Be*	12.2	8	0.0369	0.989
	Clo	*Cap*	9.8	13	0.974	0.0774

Supplementary information on the joint species distribution modelling

The R package 'trap17' including the analytical pipeline as well as data can be found in Github (aminorberg/trap17-pkg/nat-comms_publ) as well as archived in Zenodo (10.5281/zenodo.4117739).

Model variants

We fitted in total three joint species distribution model (JSDM) variants (see Table 1 in main text). We applied a JSDM framework 'Hierarchical Modelling of Species Communities' (HMSC, Ovaskainen *et al.* 2017). The framework is implemented as an R package 'Hmsc' (version 3.0-6; Tikhonov et al. 2020) and we used R version 4.0.0 [11]. All the model variants (introduced below in more detail) included the presences and absences (1/0) of the five focal viruses as response variables, but they differed in whether or not the host plant genotype was included in the fixed environmental covariates and whether the latent variable random effects at the level of individual host plants were allowed to covary with the host plant genotype or not. The structure of the JSDM is explained in detail by Ovaskainen *et al.* (2017), except regarding model variant 3 with genotype-dependent latent variables, details of which are described by Tikhonov *et al.* (2017).

In **model variant number 1** we include the all the focal explanatory variables of this study except host plant genotype, namely wild population context (categorical variable with four classes), signs of herbivory (categorical variable with two classes) and host plant size (continuous variable). We also include the latent variable random effects at the level of individual host plants (320 units). With this variant we answer the questions 'Can we explain the virus occurrences as a function of the focal environmental covariates, excluding the effects of the host plant genotype? And after

accounting for the aforementioned effects, can we find signal of species co-occurrences at the level of individual host plants?'

Model variant number 2 is otherwise identical to variant 1, except that it includes also the host plant genotype (categorical variable with four classes) as a fixed explanatory variable. Thus, as we compare this variant to the variant 1, we can answer the following questions: 'Can we explain the virus occurrences *better* as a function of not only the focal environmental covariates but also the host plant genotype? And after accounting for the aforementioned effects, can we find signal of species co-occurrences at the level of individual host plants?'

Model variant number 3, our final variant, is identical to variant 2, except that we allow the latent variables at the level of the individual host plants to covary with the host genotype (80 individual host plants per genotype). With this variant we can answer the following questions: 'Can we explain the virus occurrences as a function of the focal environmental covariates and the host plant genotype? And after accounting for the aforementioned effects, can we find signal of species co-occurrences at the level of individual host plants when these co-occurrences are allowed to differ between genotypes?'

For each model variant, we ran two independent Markov chain Monte Carlo (MCMC) chains of length 300 000 iterations, of which the first 200 000 were discarded and the resulting iterations were thinned to every 100th iteration, resulting in 1000 posterior samples. Details regarding the MCMC sampling are described in Ovaskainen *et al.* (2017). We used the default priors of the 'Hmsc' package (version 3.0-6). In addition, to test the effect of different prior parameters for the latent variables, we fitted the same set of models with two alternative priors. First, we used the default of $\alpha = (50{,}50)$,

which imposes a lot of shrinkage. Second, we used $\alpha = (3,3)$, which imposes much less shrinkage, but as a trade-off increases the risk of model overfitting. We compared the models with different priors and equal MCMC lengths. The main motivation for this was to see if we could detect more signals of biotic interactions (i.e. significant residual correlations) between viruses if we allow the model to fit the latent variables with less shrinkage.

Based on the explanatory and predictive powers of the models, we could not see a clear improvement in the model performance when using less-shrinking prior parameters. Most importantly, we did not see any major changes in the significant residual correlations. For model variants 2 and 3 with less-shrinking prior parameters we detected one virus-virus association with strong statistical support: betapartitivirus and caulimovirus. From this we conclude that there is potential for detecting significant residual associations between species, but our dataset does not provide enough signal for making any strong conclusions regarding this.

Comparison of model performance

We investigated the performance and selected the best variant of the models based on their explanatory and predictive power. We compared the model variants based on measures calculated from predictions made using all the data for training and then predicting the same response data with the fitted model (explanatory power), as well as predictions made with a 10-fold cross-validation, i.e. we predicted all the folds independently, with a separate model trained with the rest of the data, and used these predictions for calculating the performance measures (predictive power). Our model variants correspond to three competing representations of the system, and we want to compare them in order to see which one captures the signal in our data the best, not only in terms of explaining the

data the best, but also in terms of being general enough to produce adequate predictions for validation data.

To evaluate the performance of the variants at the level of host plants (320 units of observation), we calculated the Tjur R^2 coefficients of determination [13], with the built-in evaluation function 'evaluateModelFit' of the package 'Hmsc'. We also wanted to evaluate the performance of the models at a higher spatial level, so we calculated Spearman's ρ coefficients of correlation (with base function 'cor', method = "spearman") between the predicted and true occurrences of the virus species at the level of genotypes and populations, resulting in $4 \times 4 = 16$ units of observation. The results are presented in Table S1 below.

Supplementary table 4. Explanatory and predictive power of the model variants for all species.

		Explanatory power		Predictive power	
Model variant	Species	Tjur R^2	Spearman's ρ	Tjur R^2	Spearman's ρ
	Clo	0.080	0.43	0.034	0.69
	Be	0.071	0.42	0.026	0.56
1	*Cap*	0.18	0.64	0.15	0.88
	Cau	0.028	0.27	-0.0010	0.56
	En	0.011	0.14	-0.0075	0.38
	Clo	0.17	0.50	0.024	0.71
	Be	0.11	0.52	0.023	0.76
2	*Cap*	0.51	0.78	0.49	0.90
	Cau	0.042	0.39	0.0083	0.65
	En	0.021	0.32	-0.0076	0.72
3	*Clo*	0.15	0.49	0.027	0.70

	Be	0.095	0.51	0.023	0.74
	Cap	0.52	0.79	0.49	0.90
	Cau	0.054	0.38	0.0084	0.65
	En	0.027	0.34	-0.0066	0.71

Convergence of the MCMC chains

We fitted two independent MCMC chains for each variant. We investigated the mixing of the parameters by visually observing the trace plots, as well as by formally checking the potential scale reduction factor (psfr) values [14]. For our best performing variant number two, the lowest point estimate for the psfr was 1.0 and the highest was 1.3, and 39 out of the total 45 parameters had a psfr value between 0.99 and 1.1, and all of the point estimates were below their corresponding upper confidence limits. Hence, we concluded that our MCMC chains have converged sufficiently.

References

1. Roumagnac, P. *et al.* Alfalfa Leaf Curl Virus: an Aphid-Transmitted Geminivirus. *J. Virol.* **89**, 9683–9688 (2015).

2. Susi, H. *et al.* Genome sequences of a capulavirus infecting Plantago lanceolata in the Åland archipelago of Finland. *Arch. Virol.* **162**, 2041–2045 (2017).

3. Susi, H., Filloux, D., Frilander, M. J., Roumagnac, P. & Laine, A.-L. Diverse and variable virus communities in wild plant populations revealed by metagenomic tools. *PeerJ* **2019**, e6140 (2019).

4. Fuchs, M. *et al.* ICTV virus taxonomy profile: Closteroviridae. *J. Gen. Virol.* **101**, 364–365 (2020).

5. Virus Taxonomy: Classification and nomenculture of viruses. Ninth report of the international committee of taxonomy of viruses. *ICTV* **Elsevier A**, (2012).

6. Vainio, E. J. *et al.* ICTV virus taxonomy profile: Partitiviridae. *J. Gen. Virol.* **99**, 17–18 (2018).

7. Griffith, D. M., Veech, J. A. & Marsh, C. J. cooccur : Probabilistic Species Co-Occurrence Analysis in R. *J. Stat. Softw.* **69**, 1–17 (2016).

8. Veech, J. A. A probabilistic model for analysing species co-occurrence. *Glob. Ecol. Biogeogr.* **22**, 252–260 (2013).

9. Ovaskainen, O. *et al.* How to make more out of community data? A conceptual framework and its implementation as models and software. *Ecol. Lett.* **2**, 561–576 (2017).

10. Tikhonov, G. *et al.* Hmsc: Hierarchical Model of Species Communities. R package version 3.0-6. (2020).

11. R Core Team. R: A Language and Environment for Statistical Com- puting. R Foundation for Statistical Computing, Vienna, Austria. URL http://www.R-project.org. (2020).

12. Tikhonov, G., Abrego, N., Dunson, D. & Ovaskainen, O. Using joint species distribution models for evaluating how species-to-species associations depend on the environmental context. 443–452 (2017). doi:10.1111/2041-210X.12723

13. Tjur, T. Coefficients of Determination in Logistic Regression Models—A New Proposal: The Coefficient of Discrimination. *Am. Stat.* **63**, 366–372 (2009).

14. Gelman, A. & Rubin, D. B. Inference from Iterative Simulation Inference from Iterative Simulation Using Multiple Sequences. *Stat. Sci.* **7**, 457–472 (1992).
