# Supplementary material for: Intraspecific host variation plays a key role in virus community assembly
Source: Nat Commun. 2020 Nov 5;11:5610. doi: 10.1038/s41467-020-19273-z (PMC7644774; doi:10.1038/s41467-020-19273-z)
Supplement: Supplementary file 3 — Reporting Summary [file 41467_2020_19273_MOESM3_ESM.pdf]

nature research

Corresponding author(s): Suvi Sallinen

Last updated by author(s): Oct 5, 2020

Reporting Summary

Nature Research wishes to improve the reproducibility of the work that we publish. This form provides structure for consistency and transparency in reporting. For further information on Nature Research policies, see Authors & Referees and the Editorial Policy Checklist .

Statistics

For all statistical analyses, confirm that the following items are present in the figure legend, table legend, main text, or Methods section.

n/a	Confirmed	
☐	☒	The exact sample size (*n*) for each experimental group/condition, given as a discrete number and unit of measurement
☐	☒	A statement on whether measurements were taken from distinct samples or whether the same sample was measured repeatedly
☐	☒	The statistical test(s) used AND whether they are one- or two-sided<br>*Only common tests should be described solely by name; describe more complex techniques in the Methods section.*
☐	☒	A description of all covariates tested
☐	☒	A description of any assumptions or corrections, such as tests of normality and adjustment for multiple comparisons
☐	☒	A full description of the statistical parameters including central tendency (e.g. means) or other basic estimates (e.g. regression coefficient) AND variation (e.g. standard deviation) or associated estimates of uncertainty (e.g. confidence intervals)
☐	☒	For null hypothesis testing, the test statistic (e.g. *F*, *t*, *r*) with confidence intervals, effect sizes, degrees of freedom and *P* value noted<br>*Give P values as exact values whenever suitable.*
☐	☒	For Bayesian analysis, information on the choice of priors and Markov chain Monte Carlo settings
☐	☒	For hierarchical and complex designs, identification of the appropriate level for tests and full reporting of outcomes
☐	☒	Estimates of effect sizes (e.g. Cohen's *d*, Pearson's *r*), indicating how they were calculated

Our web collection on statistics for biologists contains articles on many of the points above.

Software and code

Policy information about availability of computer code

Data collection	No software was used.
Data analysis	We used the R package 'Hmsc' (version 3.0-4, Tikhonov et al. 2019) and R version 3.6.2 (R Core Team 2019). The R package including the analytical pipeline and data are openly available in Github via https://github.com/aminorberg/trap17-pkg.

For manuscripts utilizing custom algorithms or software that are central to the research but not yet described in published literature, software must be made available to editors/reviewers. We strongly encourage code deposition in a community repository (e.g. GitHub). See the Nature Research guidelines for submitting code & software for further information.

Data

Policy information about availability of data

All manuscripts must include a data availability statement. This statement should provide the following information, where applicable:

- Accession codes, unique identifiers, or web links for publicly available datasets
- A list of figures that have associated raw data
- A description of any restrictions on data availability

We provide a data availability statement in the manuscript. The data supporting our results and R codes used to analyse the data are available in Github and accessible via https://github.com/aminorberg/trap17-pkg.

Field-specific reporting

Please select the one below that is the best fit for your research. If you are not sure, read the appropriate sections before making your selection.

Ecological, evolutionary & environmental sciences study design

All studies must disclose on these points even when the disclosure is negative.

Study description	We tested whether host genotype explains within-host virus communities by performing an experiment, where four Plantago lanceolata plant individuals were cloned into 80 replicates each (each set is called genotype), resulting in 320 plants total. These plants were taken to the Åland Islands and placed into four naturally occurring P. lanceolata populations during seasonal virus epidemics. Each study population had 20 replicates per genotype, total 80 plants per population. From the experimental plants, we sampled leaves twice (two weeks after exposure and seven weeks after exposure) to detect presence/absence of five viruses, known to infect P. lanceolata in the Åland Islands, with specific PCR-primers. Per plant, virus presence/absence data was pooled so that each plant had one virus community, and the virus communities that had occupied the plants over the epidemic season were used as the response variable. In addition, symptoms of herbivory (presence/absence) and plant size were measured. We fitted multiple models ranging from an intercept-only to a full model with all variables with virus communities as the response variable and a set of the following explanatory factors: host genotype (four levels), wild population (four levels), signs of herbivory (presence or absence), plant individual (latent variable).
Research sample	Four greenhouse-grown Plantago lanceolata plant individuals that originate from the Åland Islands were cloned into 80 replicates for our experiment testing whether host genotype explains within-host virus communities. The four individuals were chosen because they originate from populations far from each other and therefore gene flow between these populations is not expected. Hence, these plants are expected to represent different genotypes. These plants were also found free of the focal viruses when tested. To detect viruses with specific PCR-primers, we collected leaf samples from each cloned experimental plant from leafs with similar age and with similar leaf area in order to have comparable samples.
Sampling strategy	DNA- and RNA-samples, sized 1cm^2 and 3cm^2 respectively, were collected from each experimental plant twice: first after two weeks of exposure and again seven weeks of exposure (five weeks in between) to ensure having both early season and later season infections included. Herbivory symptoms were recorded from each plant at the same time with DNA- and RNA-sampling. No statistical sample size calculation was done, we used the maximum feasible number of plants and populations. One leaf sample per plant is customary in this type of studies.
Data collection	Data was collected as follows: length and width (cm) of the longest leaf of each plant were measured with a ruler in the first week of the experiment on 22nd, 23rd, 24th, and 25th of May 2017 by Suvi Sallinen and field assistants Mikko Jalo, Pauliina Hyttinen, and Vanja Milenkovic. RNA and DNA-samples, and herbivory data were collected in the second week of exposure and seventh week of exposure by Suvi Sallinen, and field assistants Mikko Jalo and Pauliina Hyttinen. Laboratory work was performed after the experiment had finished. DNA was extracted at the Institute of Biotechnology at the University of Helsinki, RNA was extracted by Suvi Sallinen and laboratory technicians Laura Häkkinen and Pauliina Hyttinen. Virus community data was compiled from PCR-reactions using specific primers and resolving PCR-amplicons on agarose gel by Suvi Sallinen.
Timing and spatial scale	The experiment began when the experimental plants were taken to their study populations. Experimental plants were taken to their study populations so that one study site was established per day: on 22nd, 23rd, 24th, and 25th of May 2017. Data were collected from each plant individual: they were sampled for viruses (DNA and RNA) and herbivory on the same order and weekday as they were taken to the sites. This was done twice: between 5th and 8th of June 2017, and again between 11th and 24th of July. Data from these time points was pooled and the resulting virus communities of the epidemic season was used in the analysis.
Data exclusions	No data was excluded.
Reproducibility	Protocols used, origin of plant material, and locations of the study sites have been reported. R code used to analyse the data is publicly available in Github (aminorberg/trap17-pkg).
Randomization	Equal number of experimental plants of each genotype were placed to each of the study populations. Experimental plants were randomly spread among the natural vegetation in the study populations. The plants were randomized again three times per week for the whole study period to avoid within-population spatial effects.
Blinding	Experimental plants were named with a running number in the field. In the laboratory, the PCR-result was interpreted separately before it was combined with other information of the sample, such as experimental plant ID, population or/and genotype.

Did the study involve field work? ☒ Yes ☐ No

Field work, collection and transport

Field conditions	Measuring abiotic variation was not relevant for this study. Within study populations, plants were exposed to similar conditions.
Location	Experimental plants were divided into four populations in the Åland Islands, SW Finland. Population ID-numbers and coordinates for these study populations are : population 877 (110596.8, 6687221), population 9031 (133693.8, 6699500), population 433 (125228.9, 6684310), and population 3302 (110620.2, 6714274).
Access and import/export	Study populations were chosen so that the field work would not disturb local life: meadows were outside of residential areas and mostly surrounded by forest and/or sea shore. Furthermore, the sites were not located on cultivated or protected areas. For one site that needed driving through a property, permission for doing so was asked.
Disturbance	Study populations were chosen so that field work would not disturb local life or natural habitats (e.g. meadow surrounded by forest). Populations had a road connected to them for easy access and parking. Field work was conducted during normal

business hours.

Reporting for specific materials, systems and methods

We require information from authors about some types of materials, experimental systems and methods used in many studies. Here, indicate whether each material, system or method listed is relevant to your study. If you are not sure if a list item applies to your research, read the appropriate section before selecting a response.

Materials & experimental systems

n/a	Involved in the study	
☒	☐	Antibodies
☒	☐	Eukaryotic cell lines
☒	☐	Palaeontology
☒	☐	Animals and other organisms
☒	☐	Human research participants
☒	☐	Clinical data

Methods

n/a	Involved in the study	
☒	☐	ChIP-seq
☒	☐	Flow cytometry
☒	☐	MRI-based neuroimaging
